# On-the-Fly Short-Pulse R2R Laser Patterning Processes for the Manufacturing of Fully Printed Semitransparent Organic Photovoltaics

**DOI:** 10.3390/ma15228218

**Published:** 2022-11-18

**Authors:** Christos Kapnopoulos, Alexandros Zachariadis, Evangelos Mekeridis, Spyros Kassavetis, Christoforos Gravalidis, Argiris Laskarakis, Stergios Logothetidis

**Affiliations:** 1 Lab for Thin Films—Nanobiomaterials—Nanosystems & Nanometrology (LTFN), Department of Physics, Aristotle University of Thessaloniki, 54124 Thessaloniki, Greece; 2 Organic Electronic Technologies (OET), 20 km Thessaloniki-Tagarades Road, 57001 Thessaloniki, Greece

**Keywords:** laser pattering, laser ablation, fully printed, semitransparent module, organic photovoltaics, roll-to-roll

## Abstract

Ultrafast laser patterning is an essential technology for the low-cost and large area production of flexible Organic Electronic (OE) devices, such as Organic Photovoltaics (OPVs). In order to unleash the potential of ultrafast laser processing to perform the selective and high precision removal of complex multilayers from printed OPV stacks without affecting the underlying nanolayers, it is necessary to optimize its parameters for each nanolayer combination. In this work, we developed an efficient on-the-fly picosecond (ps) laser scribing process (P1, P2 and P3) using single wavelength and single step/pass for the precise and reliable in-line patterning of Roll-to-Roll (R2R) slot-die-coated nanolayers. We have investigated the effect of the key process parameters (pulse energy and overlap) on the patterning quality to obtain high selectivity on the ablation of each individual nanolayer. Finally, we present the implementation of the ultrafast laser patterning process in the manufacturing of fully R2R printed flexible semitransparent OPV modules with a 3.4% power conversion efficiency and 91% Geometric Fill Factor (GFF).

## 1. Introduction

Organic Photovoltaics (OPVs) are among the most promising solutions in the competitive market of emerging photovoltaic technologies today. The competitive advantage that OPVs offer is the fabrication on flexible polymer substrates by additive fabrication methods (as printing), enabling energy harvesting functionalities to numerous existing and new consumer products, as well as to large and complex shaped surfaces (e.g., in buildings, roofs, automotive and transportation, greenhouses, marine, etc) [1,2,3,4]. Some of their unique advantages include their thin film form factor, lightweight nature, conformability to curved surfaces and capability for fabrication by cost-effective production processes, such as Roll-to-Roll (R2R) printing, digital fabrication in custom-shaped devices, use of environmentally friendly materials and solvents and recyclability [3,5,6,7]. Although the majority of the research efforts from the scientific and technological communities targets the increase in the efficiency and stability of OPV devices, in normal and inverted architectures, mainly in a laboratory scale by using wet methods, such as spin coating and small-scale printing, the use of R2R printing in lab and large scale processes is rapidly expanding [8,9,10,11,12,13,14].

Moreover, a prerequisite for the commercialization of OPV devices in existing and new applications and products is the capability to manufacture large area OPVs on flexible substrates with reproducible properties (e.g., optical, electrical), homogeneity in thickness and structure, as well as stable performance over large areas [8,15,16,17,18,19]. The thickness and properties homogeneity over the whole printed area can contribute to the minimization of the ohmic losses and to the increase in current output leading to high device efficiency. This still remains as a major scientific and technological challenge because it requires the optimization of the material solution preparation, as well as of the printing and web roll treatment parameters (e.g., curing, annealing, etc.).

The fabrication of fully printed OPV devices by a R2R pilot line includes the sequential printing of the different nanolayers on a flexible substrate to form the inverted OPV device architecture [20]. During this process, the printed nanolayers must be patterned with high precision and large-scale repeatability in order to form a series of OPV devices connected to a module [21,22,23,24,25,26]. However, the conventional patterning methods, such as photolithography, include several etching steps, chemicals and optical masks, whereas they cannot be used in line with the manufacturing process. Therefore, ultrafast pulsed laser processes have an enormous potential to revolutionize the R2R manufacturing of Organic Electronic (OE) devices, since they can provide high quality and reproducible patterns by selectively removing thin film layers [21,27,28]. The short-pulse widths (typically in the range of 2–10 ps) ensures the minimization of the thermal effects on the subsequent layers.

The manufacturing of an OPV module combines three laser patterning processes (P1, P2, P3), which are required for the separation and monolithic interconnection of the individual OPV cells. More specifically, these processes are the P1 for the removal of the bottom transparent electrode; P2 for the removal of the electron transport layer (ETL), photo active layer (PAL) and hole transport layer (HTL); and finally P3 for the removal of the HTL and transparent top electrode [21,27,29,30,31,32,33,34,35]. The best scribing quality and high-power conversion efficiencies are accomplished with femtosecond (fs) laser systems, which are extremely sensitive to the industrial environment and apply multiple passes for a single P2 scribing process due to reduced ablation efficiency, which cannot be upscaled to an R2R scale. The combination of on-the-fly R2R laser patterning with an industrial picosecond (ps) laser system using a single wavelength and single pass for all scribing processes has not been achieved yet.

Key requirements for the successful laser patterning of OPV nanolayers are: (a) selective layer patterning with the complete removal of certain layers, (b) no layer residues on the surface, (c) no substrate layer damage (thermal, mechanical, etc.), (d) good quality laser scribed edge control with minimum edge burr if possible <100 nm (i.e., less than the typical layer thickness) to prevent short circuits between the individual stack layers, (e) no laser redeposited debris on the surface surrounding the scribes as it can have a detrimental effect on the device performance.

Finally, the reliable manufacturing and performance of large-scale OPV modules demand a homogenous thickness distribution across the area of the printed layers along with the stable composition of the donor: acceptor (D-A) organic semiconductors. Additionally, effective laser scribing requires knowledge of the photoactive layer’s optical constants (e.g., absorption coefficient) and therefore the laser penetration depth and a stable effective optical response of the materials across the printed area, which can be affected by the overall composition and diversities in the case of blended layers.

In this work, we present an effective methodology for the optimization of an upscaled ps pulsed laser patterning process for the reliable fabrication of high-efficiency R2R-printed OPVs on flexible substrates, combining in-line Spectroscopic Ellipsometry (SE) for the optical characterization of the printed nanolayers. The reported methodology employs a single laser source harmonic for all (P1, P2 and P3) for the on-the-fly laser ablation processes that simplify the R2R process by avoiding laser alignment and focusing steps due to laser wavelength changes. Thus, this is focused to a high-throughput industrial R2R production of OPV modules with an enormous potential for fast market commercialization.

## 2. Materials and Methods

### 2.1. Experimental Details

**Figure 1:**
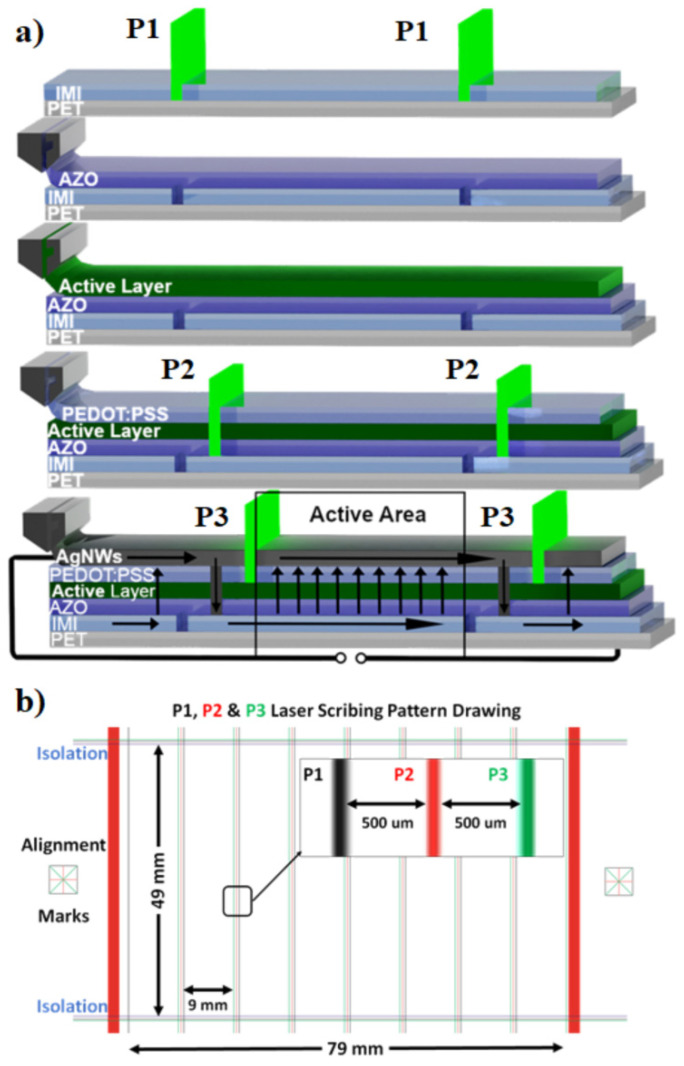
(**a**) Schematic of OPV device structure with P1, P2 and P3 scribes and (**b**) laser scribing pattern used for the manufacturing of OPV modules.

The OPV devices of this work were fully printed on commercially supplied PolyEthylene Terephthalate (PET) flexible web rolls (DuPont Teijin Films, Dumfries, Scotland, UK) of 15 cm width, precoated with an IMI film (sandwiched Indium-Tin-Oxide (ITO)/Ag/ITO). Commercially supplied Aluminum doped Zinc Oxide (AZO) ink (Avantama AG, Stäfa, Switzerland) was printed onto the PET/IMI to serve as the electron transport layer (ETL), whereas poly-3,4-ethylenedioxy-thiophene:poly (styrenesulfonic-acid) (PEDOT: PSS) (Heraeus Deutschland GmbH & Co. KG, Leverkusen, Germany) was used as the hole transport layer (HTL), and silver NWs (AgNWs) ink as the top contact electrode (Figure 1a).

The photoactive layer of the printed OPVs consisted of a polymer donor (D) and a fullerene derivative acceptor (A) provided in the form of a ready-to-use commercial ink (OET-D2A1) from OET PC. The OPV single cells and the Sheet-to-Sheet (S2S) OPV modules were fully printed by the use of a roll coater (FOM Technologies, Copenhagen, Denmark) and the R2R OPV modules were fully printed in the R2R pilot line of the Nanotechnology Lab LTFN, Aristotle University of Thessaloniki (www.ltfn.gr). The OPV nanolayers were printed by slot die coating with a printing speed of 1 m/min, whereas they were sequentially dried in a hot air oven for 3 min at 120 °C, simulating a continuous R2R manufacturing process where all the layers are printed sequentially in a single pass to form the complete OPV stack.

The optical properties of the laser-scribed nanolayers were investigated by Visible-far ultraviolet SE in both ex situ and in-line configurations. The ex situ measurements were taken by an SE (UVISEL, Horiba, Palaiseau, France) in which we performed the transmittance measurements at 90° configuration in the 1.5–6.5 eV spectral range. For the scanning SE measurement of the photoactive layer, an in-line SE system (UVISEL, Horiba, Palaiseau, France) adapted on the R2R pilot line was used, and the measurements were performed at 32 specific photon energies in the same spectral region.

The surface quality of the printed OPV nanolayers was investigated by a CX23 Upright Microscope from Olympus equipped with 4×, 10× and 40× optical lenses and a 5 M EP50 digital camera. Field-emission scanning electron microscopy (FE-SEM) and the corresponding electron dispersive X-ray spectroscopy (EDX) investigations were performed with a NEON 40 (Carl Zeiss Microscopy GmbH, Jena, Germany) Scanning Electron Microscope operating at an accelerating voltage of 1.0 kV. The photo current of the fabricated OPV devices was measured under air mass AM 1.5 G solar illumination and 100 mW/cm^2^ of irradiation using a Newport Oriel Solar Simulator (91191) where the intensity was calibrated using a KG filtered silicon reference cell.

### 2.2. Laser Processes

The laser patterning processes (P1, P2, P3) for the manufacturing of OPV modules are shown in Figure 1a and reflect the individual stack layers, which are typically in the range of 30 to 280 nm thick. The black arrows indicate the photocurrent path of an illuminated OPV module under short circuit conditions to illustrate how the P1, P2 and P3 scribes control the current path to maximize photovoltaic cell efficiency. The distance between the P1 to P3 scribes is called the “dead area” of the cell since this area does not contribute to the generation of electricity. One of the advantages of laser scribing is that significantly reduced dead areas can be achieved compared with large area printing methods [27]. The ratio between the area that produces a photo current (photoactive area) and the total area of the module is referred to as the Geometric Fill Factor (GFF). Figure 1b shows the laser patterning drawing used to form the OPV modules. All the laser scribing processes were performed on the fly on the moving web in the R2R line and therefore relatively large gaps of 500 μm, comparing the best values (80 μm) reported in the literature [28], between the P1/P2 and P2/P3 scribes were selected due to the limitations of the web handling systems used in the R2R pilot line. The edge guiding systems can control the lateral displacement of the web with an accuracy of ±100 μm on the next roller after the system. Such limitations are very important to take into account during the optimization of the on-the-fly R2R laser scribing process.

The laser system used was installed in a two-meter-long cabinet on the R2R pilot line and was equipped with a picosecond laser (ps) source with a pulse duration < 10 ps and a beam quality of the system M2 < 1.5 (EKSPLA uab, Vilnius, Lithuania). A picosecond laser pulse is comparable to electron–photon relaxation and is short enough for “cold” ablation. Moreover, ps pulses are short enough for very precise and stress-free micromachining. The laser beam is guided to the web through digital encoder scanner system with a 300 × 300 mm field of view. We have used a single wavelength of 532 nm for all the laser patterning processes (P1, P2, P3) because all the processed nanolayers are absorbing in this wavelength. Moreover, with this simplified methodology, we avoid the wavelength changes among adjacent P1, P2 and P3 processes, which require numerous laser alignments and focusing steps. Therefore, we enable cost-effective on-the-fly R2R laser patterning manufacturing processes by reducing laser equipment complexity and cost.

An x–y stage equipped on the R2R laser system with a vacuum table for the placement of samples was used for the optimization of short-pulse laser patterning processes. The x–y stage has a 200 mm travel distance on the x direction in order to be able to simulate the movement of the web below the field of view of the scanners, enabling the optimization of the laser scribing process on A4 size samples in an R2R environment [36]. The laser beam is focused on the samples using a *z*-axis moving stage for the adjustment of the scanner’s height. The laser system has two camera systems, one for projecting the scribed area for quality control and one for image recognition for registration control while running in R2R or S2S processes. The system is equipped with a suction system using special filters in order to remove the scribing residues that are formed right after the scribing process [30,37].

For the optimization and manufacturing of the OPV modules, sequential printing and laser patterning processes were performed. In more details, as the first step the P1 laser scribing process was applied on the PET/IMI substrate. This scribing process was used to segment the first conductive layer PET/IMI into adjacent, electrically isolated stripes. The next step was the sequential printing of the ETL, PAL and HTL followed by the P2 laser scribing process targeting to create a clean opening down to the IMI layer for the top electrode to create an interconnection between the OPV cells. The next is the printing of the top electrode and the P3 laser scribing process for the separation of the OPV cells as shown in Figure 1. As a final step, a laser isolation scribing process took place to separate the sequential modules produced on the 1 m length web.

## 3. Results and Discussion

### 3.1. Thin Film Properties

The efficiency and reliability of the film’s laser ablation is directly correlated with the thickness and the optical properties of the involved materials. In principle, light must penetrate through the films structure and reach the corresponding interfaces for each ablation process (P1, P2 and P3). The determination of the thickness and the optical properties of the OPV nanolayers was performed by SE measurements. The measured $\langle \tilde{\varepsilon }\left(\omega \right)\rangle$ were analysed by the use of a theoretical model that consisted of each layer structure sequence successively. The analysis process included the fitting of the experimental $\langle \tilde{\varepsilon }\left(\omega \right)\rangle$ spectra with the theoretical values by the determination of the minimization function *χ^2^* using the Levenberg–Marquardt algorithm [38,39]. In order to take into account, the optical response of the underlying layers, each layer sequence was measured successively and the pristine optical constants and average thickness were determined. The photoactive blended nanolayer was modelled with the use of the Tauc–Lorentz model and the layer described as a single-phase material combining the optical features and characteristics (absorption bands) of the two blend constituents [16,39,40]. Further discussion on the extended analysis procedure is out from the scope of this article and will be presented in a future work.

The thickness of the AZO, photoactive and PEDOT:PSS nanolayers was calculated at 35, 260 and 257 nm, respectively. The IMI bottom electrode consists of a multilayer stack of ITO/Ag/ITO with calculated thickness values of 30 nm (bottom ITO nanolayer), 15 nm (Ag) and 30 nm (top ITO nanolayer) [41]. The calculated optical parameters (real part n and imaginary part k) of the refractive index and the absorption coefficient α of the materials for the specific wavelength of the reported laser process (532 nm) are presented in Table 1.

The ablation of the photoactive layer is the most challenging issue of the P2 and P3 laser scribing processes because it has high thickness values in combination with a high absorption coefficient at this wavelength. In addition, the large area uniformity of the thickness of each individual printed nanolayer of the OPV stacks and plays a key role in ensuring a homogeneous absorption of the laser radiation during the patterning process. Moreover, the high thickness uniformity of the OPV nanolayers over large areas lead to more efficient photon absorption and carrier collection, enabling a better electrical performance of the OPV modules. Therefore, we investigated the thickness profile of the printed photoactive nanolayer over large areas, by in-line SE. By recording the pseudodielectric function <$\tilde{\varepsilon }$(ω)> using a spatial step of 1.6 mm across the rolling (web) direction, we measured 600 <$\tilde{\varepsilon }$(ω)> spectra per meter of web. Taking into consideration that the SE spot size is around 3 mm in length at the 70° angle of incidence of the light beam, the results report a fully measured coverage of the web.

**Figure 2:**
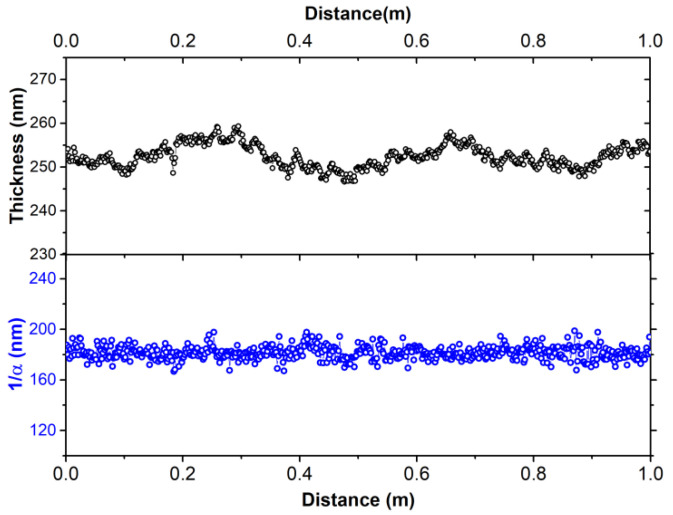
Calculated thickness (**top**) and penetration depth 1/α (**bottom**) of the photoactive layer at photon energy of 2.37 eV (wavelength of 532 nm) across the 1 m width web.

The analysis results from Figure 2 demonstrate a remarkable large area thickness uniformity of the photoactive layer with a mean thickness of 252 ± 3 nm, demonstrating a homogenous, smooth and uniform film deposition. Additionally, the negligible deviation of the 1/α value (181 ± 5 nm) reveals the homogeneity of the layer without defects, impurities or contaminations. As a result, the constant light penetration depth across the web can ensure the effective and reproducible laser patterning as well as the semitransparency and consistent light harvesting properties across the area of the OPV modules.

### 3.2. Laser Scribing

**Figure 3:**
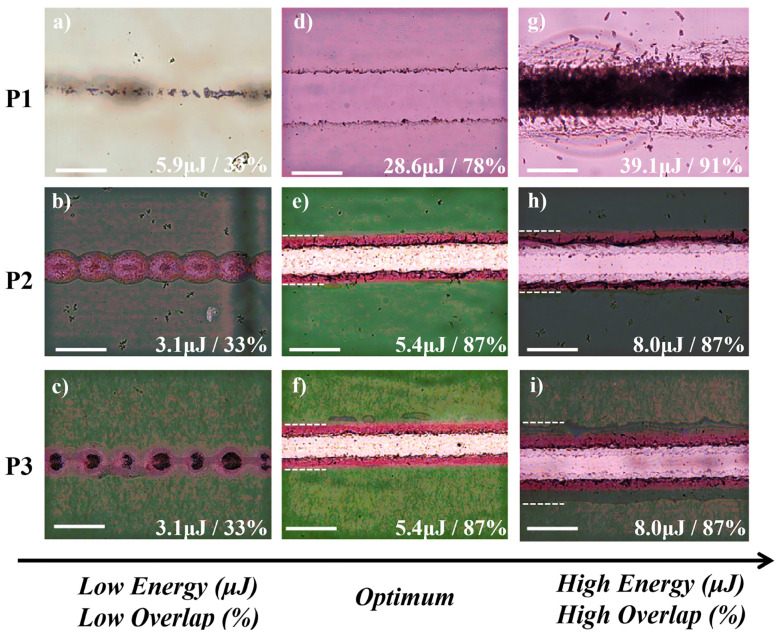
Optical microscopy images for below (**a**–**c**), optimum (**d**–**f**) and above (**g**–**i**) ablation threshold of P1, P2 and P3 processes. The white line indicates a 40 μm scale and dash line represent heat-affected zone outer boundaries.

The success of the laser scribing process is based on the optimized combination of the laser parameters based on the defined instrumentation setup. Typically, the main parameters to be optimized for the laser scribing process are the pulse energy and the pulse overlap. Representative optical microscopy images for the ablation threshold of P1, P2 and P3 laser scribing processes under various pulse energies and pulse overlap values are shown in Figure 3. Low pulse energy results in either no ablation or exclusively in thermal effects on the targeted layers. Low-pulse overlap results in noncontinuous scribe lines as indicated in Figure 3a for P1, Figure 3b for P2 and Figure 3c for P3. Yet, high pulse energy and/or high pulse overlap could cause damage on the beneath layers or create a strong heat-affected zone around the scribe line as shown in Figure 3g for P1, Figure 3h for P2 and Figure 3i for P3. Finally, the optimum pulse energy and pulse overlap laser scribing process parameters lead to a successful ablation of the targeted layers avoiding the damage of the underneath layers with a minimum heat-affected zone around the scribe line as shown in Figure 3d for P1, Figure 3e for P2 and Figure 3f for P3.

In order to reduce the trial-and-error experimental steps for the optimization of the laser scribing processes, we proceeded to the theoretical calculation of the laser ablation threshold fluence from the relationship between the squared diameter of the ablated spot r^2^ and the laser fluence F_0_ (Equation (1)). The laser ablation threshold fluence (F_Th_) is defined as the minimum laser intensity that enables material removal and is measured as energy/unit area. Typically, an operating laser process is defined as at least at 2 × F_Th_, and since this parameter is independent of a focused spot size, it is used to compare laser ablations with different focal spot sizes or laser types. F_Th_ can be calculated assuming the well-known Gaussian equation below [29,32,42,43]:

(1) FTh=F0·e−2r2w02

where F_0_ is the laser fluence, r is the laser ablated crater diameter and 2w_0_ is the focused spot size of the laser beam, defined as the distance across the centre of the beam for which the irradiance (intensity) is equal to 1/e^2^ of the maximum irradiance. Equation (1) can also be written as follows:

(2) $$2r=2w_{0}\sqrt{0.5ln\left(\frac{F}{F_{Th}}\right)}$$

**Figure 4:**
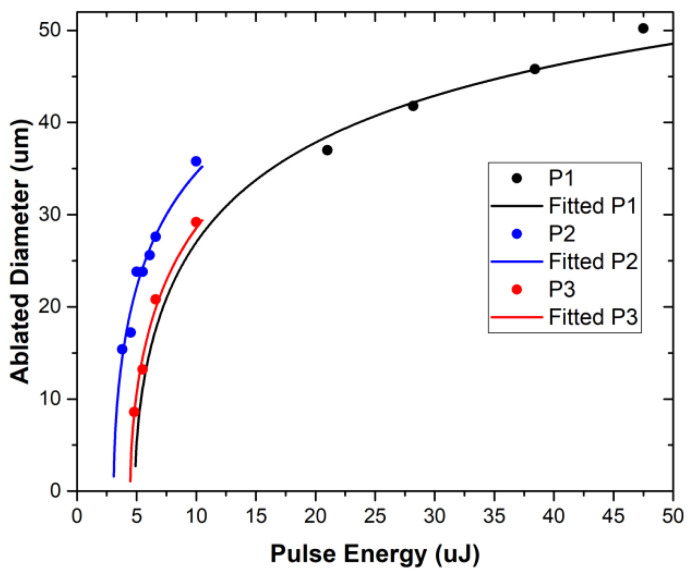
Laser ablation threshold measurement and fitted curves produced for a focused spot size of ~45 μm and a 532 nm wavelength.

According to Equation (2), the laser ablation threshold value for the laser fluence F_Th_ or laser pulse energy E_Th_ can be derived from a single 2r, and fluence (F) or energy (E) measurement at a given w_0_. However, the experimentally measured values by imaging techniques include a level of uncertainty. For the improved calculation accuracy and appreciated statistical significance of the results, Equation (2) was implemented by curve fit to calculate the F_Th_ or E_Th_ at given values of (2r) versus the laser pulse energy E (or F) plots. The crater diameter or scribe line width for a focused spot size of 45 um at 532 nm was measured by optical and SEM microscopy. Figure 4 shows the ablation diagrams that are essential to define the process window for the individual P1, P2 and P3 processes. From the curve fit presented in Figure 4, the best fitted values for the laser energy ablation threshold E_th_ are calculated to 4.87 (R^2^ = 0.93), 3.08 (R^2^ = 0.96) and 4.47 (R^2^ = 0.99) μJoule for the P1, P2 and P3, respectively. The observed deviation of the P2 ablation threshold to lower energies is ascribed to the high absorption of the polymer photoactive layer at 532 nm, and therefore the energy required for the ablation of these layers is lower than the energy required for the P1 and P3 processes, which involves laser irradiation with inorganic and metallic layers of the OPV stack. The above calculations are following the experimental data from the optical microscopy images (Figure 3a–c) and SEM images shown later in the manuscript for the optimized P2–P3 laser scribing process parameters. The calculated laser ablation thresholds were used as boundary conditions for the experimental investigation of the combined effect of pulse energy and pulse overlap.

**Figure 5:**
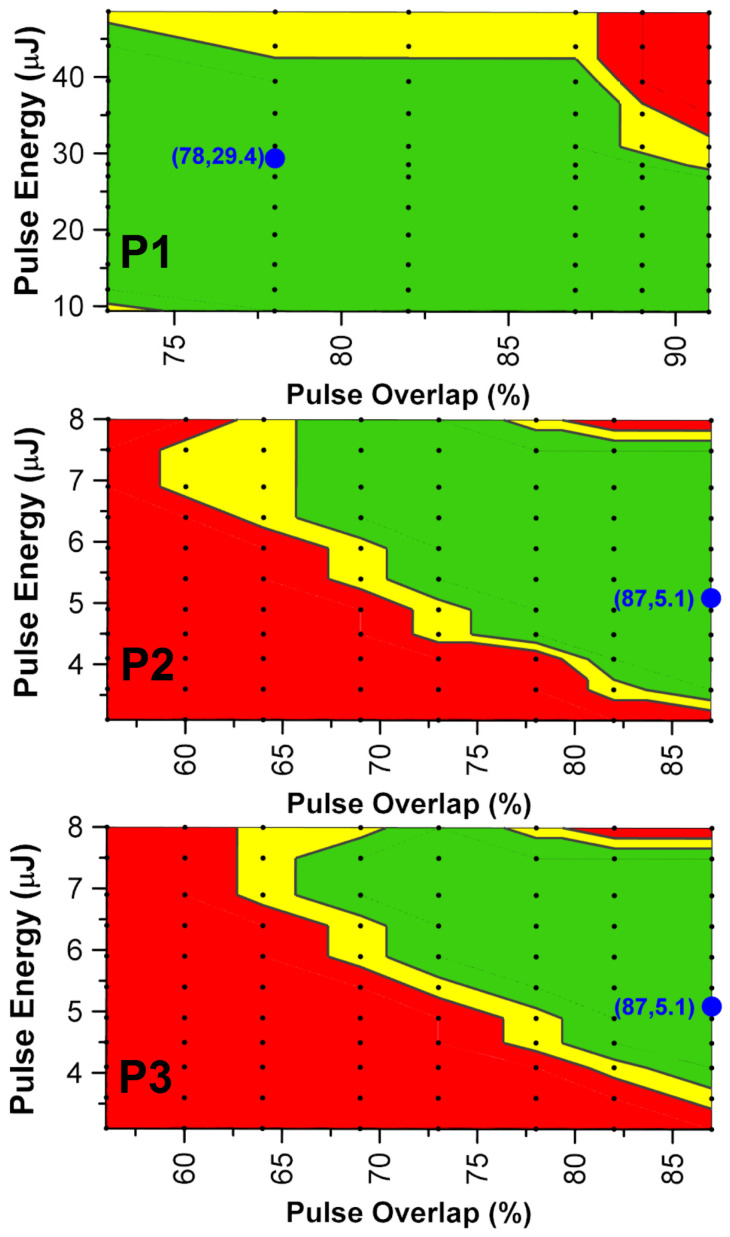
Process window map for P1, P2, P3 laser scribing. Black dots represent the parameters used for the devices.

The combined effect of the pulse energy along with the pulse overlap was experimentally investigated and is demonstrated by a traffic light system to present the process window for the different laser scribing processes (Figure 5). The green colour indicates complete, consistent scribing whereas red represents incomplete scribes or scribes with excessive damage to the underlying layers. The yellow colour refers to intermediate and doubtful quality of laser scribes. Delamination is not considered in these process window maps. The process window maps were used to identify the best process parameters that can produce the sufficient selective ablation of the printed layers with the necessary tolerance due to fluctuations of the R2R manufacturing process parameters such as the homogeneity of printing processes across the printing width and over long printing runs, deviations of the web tension and stability of the laser pulse energy over time.

**Figure 6:**
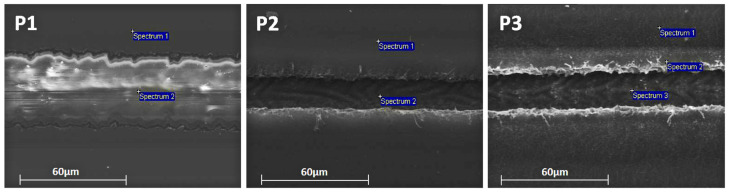
SEM images for the optimum laser parameters (blue marks indicate the EDX analysis spots).

The establishment of the selective ablation of the corresponding layers for each process was verified by SEM imaging and EDX analysis (Table 2). Figure 6 shows the optimum accomplished laser scribes for each laser process, establishing the successful removal of the desired layers without damaging the underlying structure. The P1 optimum scribing quality was achieved at a 78% pulse overlap and 29.4 μJ pulse energy. From the EDX analysis performed within the scribe line, no traces of Indium or Ag were reported, indicating the complete removal of the IMI layer. Moreover, we observed no heat-effect zone or damage delivered to the PET substrate. For P2 laser process, the best scribing quality was achieved with an 87% pulse overlap and a 5.1 μJ pulse energy, and according to the EDX analysis in the scribing line the absence of S, which is a component of the PAL as well as of the PEDOT:PSS layer, both were completely removed and at the same time the existence of In and Ag verified that the transparent electrode in still was undamaged. For the P3 laser process, the optimum laser parameters were found to be the same as for the P2, and the EDX analysis reported no S residues inside the scribe line, announcing efficient ablation not only for the top AgNw but also for the PAL/PEDOT:PSS over the AZO. Here again as for the P2 laser process from the trace of the Ag and In inside the scribe line, it can be concluded that the transparent electrode is still bridging the OPV cells in the module. Nevertheless, from the SEM images of the P2 and P3 laser process, a small delamination at the edges of the scribe line was observed due to lower pulse energy at the edges, as well as a heat effect zone due to the high focal length used in order to achieve a 300 × 300 mm field of view.

### 3.3. Fully Printed Semitransparent OPVs

Using the laser scribing process to monolithically connect the OPV device to modules, we achieved an increase in the GFF of the modules from 60% (for stripe coating) up to 91% using the fully coated surface for the printed layers and applying P1, P2 and P3 laser processes for the patterning. The attainment of this maximum GFF value is attributed to the fact that a further increase in the GFF value requires an extremely accurate displacement of the web roll during the laser process. Nevertheless, the effectiveness of the web roll displacement is affected by the resolution of the handling system used in the R2R pilot line.

**Figure 7:**
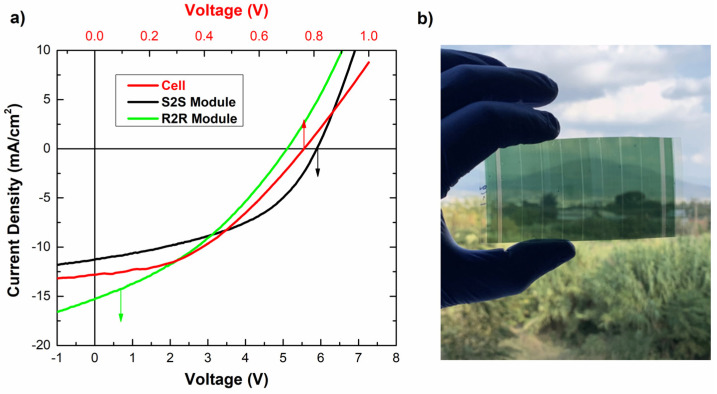
(**a**) Current density versus voltage for the fully printed semitransparent OPV cell and modules. (**b**) Photo of fully R2R printed OPV module with P1, P2 and P3 laser scribing processes achieving up to 91% Geometric Fill Factor.

In order to demonstrate the efficiency of the laser scribing process in the manufacturing of fully R2R printed semitransparent OPV modules, a comparison is presented between the performance of single OPV cells with a 1 cm^2^ active area and S2S and R2R OPV modules with different module lengths consisting of eight interconnected in-series OPV cells with a total active area up to 35.3 cm^2^. As it is declared in the J-V curves (Figure 7) and the electrical characteristics shown in Table 3, there are negligible power losses at the upscaling of the manufacturing process from a single cell to a fully printed OPV module.

In further analysis, the mean short-circuit current (J_sc_) values reported a loss of around 8.4% between the 1 cm^2^ single cells and the right interconnected S2S module cells with a 4.4 cm^2^ average active area for each cell, but for the R2R modules, we are measuring an increased J_sc_ almost 14% that can be attributed to the low fill factor of the R2R modules. The minor J_sc_ losses can be attributed to the uniform thickness profile of the active layer across the cells area and to the good quality of the laser scribing and interconnection between the cells in the modules. The mean values of the S2S modules’ open-circuit voltage (V_oc_), measured at 5.45 V as a result of the in series interconnected cells, suggests a mean value of 0.68 V per cell and a loss of around 10.5% compared with 0.76 V V_oc_ of the single cells and a loss of around 17% for the R2R modules. The V_oc_ losses in the modules can be attributed to the dissimilarity of the interconnected subshells. On the other hand, the average values of the S2S modules fill factor (FF) reported 3.3% higher values than the single cells. In combination with the small diversity of the series and shunt resistance values (R_s_, R_sh_), it can be attributed to the good quality of the laser scribing and interconnection between the cells in the modules. In contrast, the FF of the R2R printed modules showed a significant reduction mainly due to the defects created at the interfaces of the printed layers in the R2R printing processes. The number of defects in the module active area due to the contact of the coated side on the surface of the rolls of the R2R pilot line strongly affected the performance and should be the main focus of future research in order to further decrease the power losses.

Finally, the combination of the above electrical characteristics resulted in only a 15.6% reduction in the power conversion efficiency (PCE) for the 35.3 cm^2^ S2S modules and 22% for the R2R modules compared with the 1 cm^2^ single cells. These characteristics prove the reproducibility of the successful manufacturing fully R2R printed semitransparent OPV modules using on-the-fly laser scribing processes with an efficiency up to 3.4%. Τhe device transparency (without encapsulation) spread from NIR to UVA, exhibiting 29% maximum transmittance for the VIS range at 504 nm and above 40% at the NIR for wavelengths above 800 nm.

## 4. Conclusions

In this work, we investigated the on-the-fly short-pulse laser patterning processes for the R2R fabrication of large-area fully printed semitransparent flexible OPV modules. The thickness and the optical properties (real n and imaginary part k of the refractive index and the absorption coefficient a) of the OPV nanolayers was defined by SE measurements. The calculated thickness and penetration depth 1/α of the photoactive layer demonstrate a remarkable large area thickness uniformity of the photoactive layer (252 ± 3 nm), with a negligible deviation of the 1/α value (181 ± 5 nm). We theoretically calculated the ablation threshold of multiple nanolayer structures to improve the experimental investigation of the combined effect of pulse energy and pulse overlap to create the P1, P2 and P3 process windows. The selective ablation of the corresponding layers for each process was verified by SEM imaging and EDX analysis. The optimization of the P1, P2 and P3 laser scribing process revealed an innovative on-the-fly laser scribing process which uses an industrial ps laser source at a single wavelength (532 nm) in visible and performs all laser process steps in a single pass that can be applied for the in-line on-the-fly real-time R2R laser scribing of all slot-die-coated OPV nanolayers. Moreover, the upscaling of the laser scribing process from single cell to S2S modules and R2R modules was validated with 15.6 and 22% power losses, respectively. The optimized scribing parameters resulted in the manufacturing of fully printed semitransparent OPV modules exhibiting PCE up to 3.8% with minor power cell-to-module losses and a GFF of 91%. This methodology has a strong potential to improve the large area manufacturing processes of high performance and stable OE devices (OPVs, OLEDs, OTFTs, etc.) in combination with low-cost and high commercialization potential.

## Figures and Tables

**Table 1:** OPV structure, materials and their optical properties at 532 nm.

Nanolayer	*n*	k	α (1/cm)
Ag	0.129	3.214	755,692
ITO	2.372	0.005	1275
AZO	1.749	0	0
Photoactive	1.876	0.228	54,891
PEDOT:PSS	1.451	0.036	8.375
PET	1.797	0	0

**Table 2:** EDX analysis at the blue marked spots in Figure 5.

	Spec	C (%)	O (%)	S (%)	Zn (%)	Ag (%)	In (%)
P1	1	50.3	36.2	-	4.6	2.2	6.8
	2	68.8	31.2	-	-	-	-
P2	1	8.9	24.7	2.8	3.9	2	7.7
	2	54.6	29.5		4.8	2.8	8.4
P3	1	57.5	22.1	3.6	5.1	3.5	8.2
	2	57.6	22.6	3.7	4.3	3.9	7.6
	3	54.1	30.2	-	4.6	2.7	8.4

**Table 3:** Electrical characteristics of the OPV devices and modules.

	Jsc [mA/cm^2^]	Voc [V]	FF [%]	Rs [Ohm]	Rsh [Ohm]	PCE [%]	Active Area (cm^2^)	Average PCE Loss (%)
Single Cell *	12.8 (12.2)	0.76 (0.76)	42 (42.4)	30 (31)	751 (420)	4.04 (3.91)	1	0
S2S Module 8 Cells **	11.2 (11.1)	5.88 (5.45)	46 (43.8)	53 (59)	582 (515)	3.77 (3.30)	35.3	15.6
R2R Module 8 Cells ***	15.2 (14.2)	5.06 (5.02)	36 (34)	48 (58)	168 (167)	3.42 (3.05)	32	22

* The numbers in the brackets are the average values of 15 OPV Cells. ** The numbers in the brackets are the average values of 5 OPV modules. *** The numbers in the brackets are the average values of 3 R2R OPV modules.

## Data Availability

Data sharing is not applicable.
